# Variability in eukaryotic initiation factor iso4E in *Brassica rapa* influences interactions with the viral protein linked to the genome of *Turnip mosaic virus*

**DOI:** 10.1038/s41598-018-31739-1

**Published:** 2018-09-11

**Authors:** Guoliang Li, Wei Qian, Shujiang Zhang, Shifan Zhang, Fei Li, Hui Zhang, Zhiyuan Fang, Jian Wu, Xiaowu Wang, Rifei Sun

**Affiliations:** Institute of Vegetables and Flowers, Chinese Academy of Agricultural Sciences, Zhongguancun, Nandajie No. 12, Haidian District, Beijing, 100081 People’s Republic of China

## Abstract

Plant potyviruses require eukaryotic translation initiation factors (eIFs) such as eIF4E and eIF(iso)4E to replicate and spread. When *Turnip mosaic virus* (TuMV) infects a host plant, its viral protein linked to the genome (VPg) needs to interact with eIF4E or eIF(iso)4E to initiate translation. TuMV utilizes BraA.eIF4E.a, BraA.eIF4E.c, BraA.eIF(iso)4E.a, and BraA.eIF(iso)4E.c of *Brassica rapa* to initiate translation in *Arabidopsis thaliana*. In this study, the *BraA*.*eIF4E*.*a*, *BraA*.*eIF4E*.*c*, *BraA*.*eIF(iso)4E*.*a*, and *BraA*.*eIF(iso)4E*.*c* genes were cloned and sequenced from eight *B*. *rapa* lines, namely, two *BraA*.*eIF4E*.*a* alleles, four *BraA*.*eIF4E*.*c* alleles, four *BraA*.*eIF(iso)4E*.*a* alleles, and two *BraA*.*eIF(iso)4E*.*c* alleles. Yeast two-hybrid (Y2H) and bimolecular fluorescence complementation (BiFC) analyses indicated that TuMV VPg could not interact with eIF4E, but only with eIF(iso)4E of *B*. *rapa*. In addition, the VPgs of the different TuMV isolates interacted with various eIF(iso)4E copies in *B*. *rapa*. In particular, TuMV-UK1/CDN1 VPg only interacted with BraA.eIF(iso)4E.c, not with BraA.eIF(iso)4E.a. Some single nucleotide polymorphisms (SNPs) were identified that may have affected the interaction between eIF(iso)4E and VPg such as the SNP T_106_C in BraA.eIF(iso)4E.c and the SNP A_154_C in VPg. Furthermore, a three-dimensional structural model of the BraA.eIF(iso)4E.c-1 protein was constructed to identify the specific conformation of the variable amino acids from BraA.eIF(iso)4E.c. The 36^th^ amino acid in BraA.eIF(iso)4E.c is highly conserved and may play an important role in establishing protein structural stability. The findings of the present study may lay the foundation for future investigations on the co-evolution of TuMV and *eIF(iso)4E*.

## Introduction

*Potyvirus* is the largest genus of plant viruses (comprising approximately 36% of the total number of plant viruses) and causes severe economic losses in agriculture^1,2^. The shared characteristics of the *Potyviridae* family include a positive single-stranded RNA molecule with a covalently-bound viral protein linked to the genome (VPg) that is linked to the 5′-end and a 3′ poly (A) tail at the 3′-end^2,3^. *Turnip mosaic virus* (TuMV) is a member of *Potyvirus* (family *Potyviridae*) and is one of the most significant potyviruses known to infect brassicas^4,5^. The genome of TuMV is approximately 10 kb in size and has a single open reading frame (ORF) that is flanked by two untranslated regions (UTRs)^5^. The 5′ UTR includes an internal ribosome entry site^5,6^. The ORF is translated as a large polyprotein that is subsequently cleaved into at least 10 smaller functional polypeptides (P1, HC-Pro, P3, 6K1, C1, 6K2, VPg, NIa, Nib, and CP) by viral-encoded proteases^3,5,7,8^. The 6K2 and VPg proteins are involved in replication complexes on cytoplasmic membranes^5^.

Approximately 51% of resistance traits to plant viruses are dominant, 35% are recessive, and the remaining are more complex (incomplete dominance or dose-dependent)^9^. Plants have both active and passive resistance mechanisms against viruses. The active resistance mechanisms are mediated by *R* genes and/or gene silencing^9^. *R* genes are always dominant and have characteristic domains such as NBS-LRR or (CC)-NBS-LRR^10^. Generally, potyviruses are only able to encode a limited number of proteins, and therefore they depend on host factors for replication and translation and to infect the host and spread systemically^11,12^. The passive mechanisms of plant virus resistance indicate that loss, deletion, or mutation of a required host factor may cause recessive resistance to the virus^2,12,13^. Several of these resistance genes (such as *pvr1* in *Capsicum* and *retr01* and *retr02* in *Brassica rapa*) have been successfully used for decades in breeding programs as effective and stable sources of resistance^14–16^.

The majority, but not all, of the recessive resistance genes that have been characterized to date encode eukaryotic translation initiation factors (eIFs) [i.e., eIF4E, eIF(iso)4E, eIF4G, and eIF(iso)4 G], which play critical roles in potyviral infection^2,12,17^. eIFs associated with plant virus resistance are encoded by genes such as *lsp1*^18^, *pvr1/2*^14,19^, *pvr6*^19^, *nsv*^20,21^, *cum2*^22^, *cum1*^22,23^, *sbm1*^24^, *rymv-1*^25,26^, *rym4/5/6*^27,28^, *mol1/2*^29^, *pot1*^30^, *cyv-1*^31^, *cyv-2*^32^, *tsv1*^33^, *wlv*^34^, *bc-*3^35^, *retr01*^15^, *retr02*^16^, and *retr03*^36^. In plants, eIF4E and eIF4G form the eIF4F complex, and eIF(iso)4E and eIF(iso)4 G form the eIF(iso)4F complex^37,38^. These complexes are involved in the binding of the mRNA cap and ribosome recruitment in the initial steps of translation^16,18,26,39^. Some studies have indicated that the potyviral protein, VPg, is crucial for virus replication and cell-to-cell communication, as well as long-distance movement in relation to recessive resistance genes in different host species^2,40–45^. A key observation in yeast two-hybrid (Y2H) assays relating to recessive resistance is that the VPg (or its precursor, NIa) protein of some potyviruses has high affinity to eIF4E or eIF(iso)4E^46,47^. For example, Y2H and enzyme-linked immunosorbent assays revealed interactions between *Arabidopsis* eIF(iso)4E and TuMV/Tobacco etch virus (TEV) VPg^2,46^.

*Arabidopsis thaliana* possesses three eIF4E genes, one eIF(iso)4E gene, three eIF4G genes, and two eIF(iso)4G genes that act as host factors and play an important role in viral infection^37^. Mutations in eIF(iso)4E in *A*. *thaliana* result in broad-spectrum potyvirus resistance to TEV and TuMV^18,48^. In addition, TuMV VPg solely interacts with the eIF(iso)4E gene (AT5G35620), and not with any other eIF4E genes in *A*. *thaliana*^49^. Y2H assays and co-immunoprecipitation analysis suggest that the W_95_L, K_150_L, and W_95_L/K_150_E amino acid mutations in *B*. *rapa* eIF(iso)4E interrupt its interaction with TuMV VPg and its overexpression in a susceptible Chinese cabbage cultivar confers resistance to multiple TuMV strains^50^.

Three copies of *eIF4E* [*BraA*.*eIF4E*.*a*, *BraA*.*eIF4E*.*b*, and *BraA*.*eIF4E*.*c*] and three copies of *eIF(iso)4E* [*BraA*.*eIF(iso)4E*.*a*, *BraA*.*eIF(iso)4E*.*b*, and *BraA*.*eIF(iso)4E*.*c*] were identified in the TuMV-susceptible, inbred, diploid *B*. *rapa* line R-o-18^51^. In addition, some recessive resistance genes to TuMV have been identified in *B*. *rapa*, such as *retr01*^15^ and *retr02*^16^, which encode eIF(iso)4E proteins. The *retr02* gene contains a polymorphism (A/G) that results in an amino acid substitution (Gly/Asp) in the eIF(iso)4E protein that contributes to resistance/susceptibility^16^. In a further study, splice variants within the *retr02* gene produced a stop codon within exon 1 that is predicted to generate a truncated, non-functional protein, and TuMV could use copies of eIF(iso)4E at two loci, which confers a spectrum of resistance and durability^52^. Subsequently, the *Brassica juncea retr03* gene was mapped and determined to be an allele of *eIF2Bβ*, which acts as a guanine nucleotide exchange factor (GEF) for its GTP-binding protein partner eIF2 by interacting with eIF2.GTP at an early step during translation initiation, thereby conferring resistance to the TuMV isolate (ZJ) from Zhejiang Province in China^36^. Based on our previous study, TuMV-C4 VPg exclusively interacted with *BraA*.*eIF(iso)4E*.*a*, and TuMV-UK1 VPg solely interacted with *BraA*.*eIF(iso)4E*.*c*^53^. In the present study, the eIF4E (*BraA*.*eIF4E*.*a* and *BraA*.*eIF4E*.*c*) and eIF(iso)4E (*BraA*.*eIF(iso)4E*.*a* and *BraA*.*eIF(iso)4E*.*c*) alleles from eight *B*. *rapa* lines were investigated. The TuMV isolates C4 [HQ46217] from China, CDN1 [D83184] from Canada, and UK1 [NC_002509] from the UK, constituted the three representative pathotypes. The interactions of *BraA*.*eIF4E*/*BraA*.*eIF(iso)4E* and TuMV-CDN1 VPgs were analyzed by Y2H and bimolecular fluorescence complementation (BiFC) assays. Furthermore, key amino acids influencing the interaction between *BraA*.*eIF(iso)4E* and TuMV-C4/UK1/CDN1 VPgs were identified. This is the first report on the systemic relationships between eIF4E families and different TuMV VPgs. This may provide a foundation for the screening and cloning of eIF(iso)4E resistance loci, as well as systematic research into the resistance mechanisms of eIF(iso)4E to TuMV.

## Results

### Variability in eIF4E and its isoform eIF(iso)4E in *B*. *rapa*

Two copies of eIF4E (*BraA*.*eIF4E*.*a* and *BraA*.*eIF4E*.*c*) and two copies of eIF(iso)4E [*BraA*.*eIF(iso)4E*.*a* and *BraA*.*eIF(iso)4E*.*c*] could potentially complement Col-0::*dSpm* [possessing a transposon knock-out of *eIF(iso)4E*]. Thus, primers were designed to amplify the cDNAs of these copies in the eight *B*. *rapa* lines, followed by sequencing. A sequence analysis identified several variants within the *BraA*.*eIF4E*.*a* of the eight lines (the lines 80122, 80186, 80124, Chiifu, 2079, and BP058 are resistant to TuMV C4, whereas 80425 and R-o-18 are susceptible). Most of the variations were non-synonymous, although some were synonymous (nts 300, 444, 525, and 564) (Table S1). There were no differences in nucleotide/amino acid sequences among lines 80124, BP058, and 2079, which included only three differing bases/amino acids (nts 98, 164, and 638; aas 33, 55, and 213) compared to line 80186 (Tables S1 and 1). The amino acid sequences of 80122, 80425, Chiifu, and R-o-18 were the same, although there were also some synonymous nucleotide variations. Numerous variations were also identified in *BraA*.*eIF4E*.*c*, although most were synonymous (Tables S2 and 2). Notably, the nucleotide/amino acid sequences of 80122 and Chiifu were the same, as were those of 80425 and 2079. In addition, the nucleotide/amino acid sequences of 80186, BP058, and R-o-18 were the same, and differed in two bases/amino acids from 80124.

**Table 1:** Amino acids changes in *BraA*.*eIF4E*.*a*.

Line	12	21	33	40	55	112	213
80124	P	V	D	I	L	Y	E
BP058	P	V	D	I	L	Y	E
2079	P	V	D	I	L	Y	E
80186	P	V	G	I	S	Y	G
80122	A	A	D	T	L	C	E
80425	A	A	D	T	L	C	E
Chiifu	A	A	D	T	L	C	E
R-o-18	A	A	D	T	L	C	E

Notes: P, proline; A, alanine; V, valine; D, aspartic acid; G, glycine; I, isoleucine; T, threonine; L, leucine; S, serine; Y, tyrosine; C, cysteine; E, glutamic acid.

**Table 2:** Amino acid changes in *BraA*.*eIF4E*.*c*.

Line	23	35	45	182	201
80122	H	T	G	R	K
Chiifu	H	T	G	R	K
80186	H	A	G	K	R
80124	R	A	G	R	R
BP058	H	A	G	K	R
R-o-18	H	A	G	K	R
80425	H	V	T	R	K
2079	H	V	T	R	K

Notes: H, histidine; R, arginine; T, threonine; A, alanine; V, valine; G, glycine; K, lysine.

There was one G insertion at the exon 1/intron 1 junction of *BraA*.*eIF(iso)4E*.*a* in 80122, 80124, BP058, and 2079 that was predicted to result in premature protein termination (Tables S3 and 3), thereby conferring resistance to TuMV. Two splice variants in *BraA*.*eIF(iso)4E*.*a* have been reported in the 80122 line, namely 80122-1, which retained intron 1, resulting in a premature stop codon at position 234 bp and 80122-2, which has an extra G at the end of exon 1, resulting in a premature stop codon^52^. The nucleotide/amino acid sequences of 80186 and Chiifu were identical (Tables S3 and 3) and differed from the 80425 line in a single nucleotide/amino acid. Compared to 80186, R-o-18 exhibited seven nucleotide variations, which consisted of five synonymous and two non-synonymous changes. The *BraA*.*eIF(iso)4E*.*c* sequence was the same among lines 80122, 80425, 80186, 80124, BP058, 2079, and Chiifu, but differed from that of the R-o-18 line, which included five nucleotide variations consisting of one synonymous and four non-synonymous amino acid changes (Tables S4 and 4). The sequence analysis of *BraA*.*eIF4E*.*a*, *BraA*.*eIF4E*.*c*, *BraA*.*eIF(iso)4E*.*a*, and *BraA*.*eIF(iso)4E*.*c* indicated that the eIF4E genes were more variable than the eIF(iso)4E genes.

**Table 3:** Amino acid changes in *BraA*.*eIF(iso)4E*.*a*.

Line	27	108	152
80122-1	D	—	—
80122-2	D	—	—
80124	D	—	—
BP058	D	—	—
2079	D	—	—
80186	D	F	D
Chiifu	D	F	D
80425	D	F	G
R-o-18	H	Y	D

Notes: D, aspartic acid; H, histidine; F, phenylalanine; Y, tyrosine; G, glycine.

**Table 4:** Amino acid changes in *BraA*.*eIF(iso)4E*.*c*.

Line	36	52	80	150
80186	F	A	I	P
80124	F	A	I	P
BP058	F	A	I	P
80122	F	A	I	P
80425	F	A	I	P
2079	F	A	I	P
Chiifu	F	A	I	P
R-o-18	L	V	T	Q

Notes: F, phenylalanine; L, leucine; A, alanine; V, valine; I, isoleucine; T, threonine; P, proline; Q, glutarnine.

### The *B*. *rapa* eIF4E genes do not interact with TuMV-VPg

Compared with the eIF(iso)4E gene, the eIF4E genes exhibited a high level of sequence variability. Two different *BraA*.*eIF4E*.*a* alleles were identified, namely, *BraA*.*eIF4E*.*a-1* (80124, BP058, 2079, and 80186) and *BraA*.*eIF4E*.*a-2* (80122, 80425, Chiifu, and R-o-18). Four different *BraA*.*eIF4E*.*c* alleles were also detected, namely, *BraA*.*eIF4E*.*c-1* (80122 and Chiifu), *BraA*.*eIF4E*.*c-2* (80425 and 2079), *BraA*.*eIF4E*.*c-3* (80186, BP058 and R-o-18), and *BraA*.*eIF4E*.*c-4* (80124). The *BraA*.*eIF4E*.*c* sequences from 80186, 80124, BP058, and R-o-18 were highly similar, and 80186 and 80124 were selected as representative lines for further analysis. The *BraA*.*eIF4E*.*a* and *BraA*.*eIF4E*.*c* genes complemented Col-0::*dSpm*, and TuMV could possibly interact with them for replication and multiplication. To confirm this, Y2H and BiFC assays were conducted between BraA.eIF4E.a/BraA.eIF4E.c and TuMV-C4/UK1/CDN1 VPgs.

**Figure 1:**
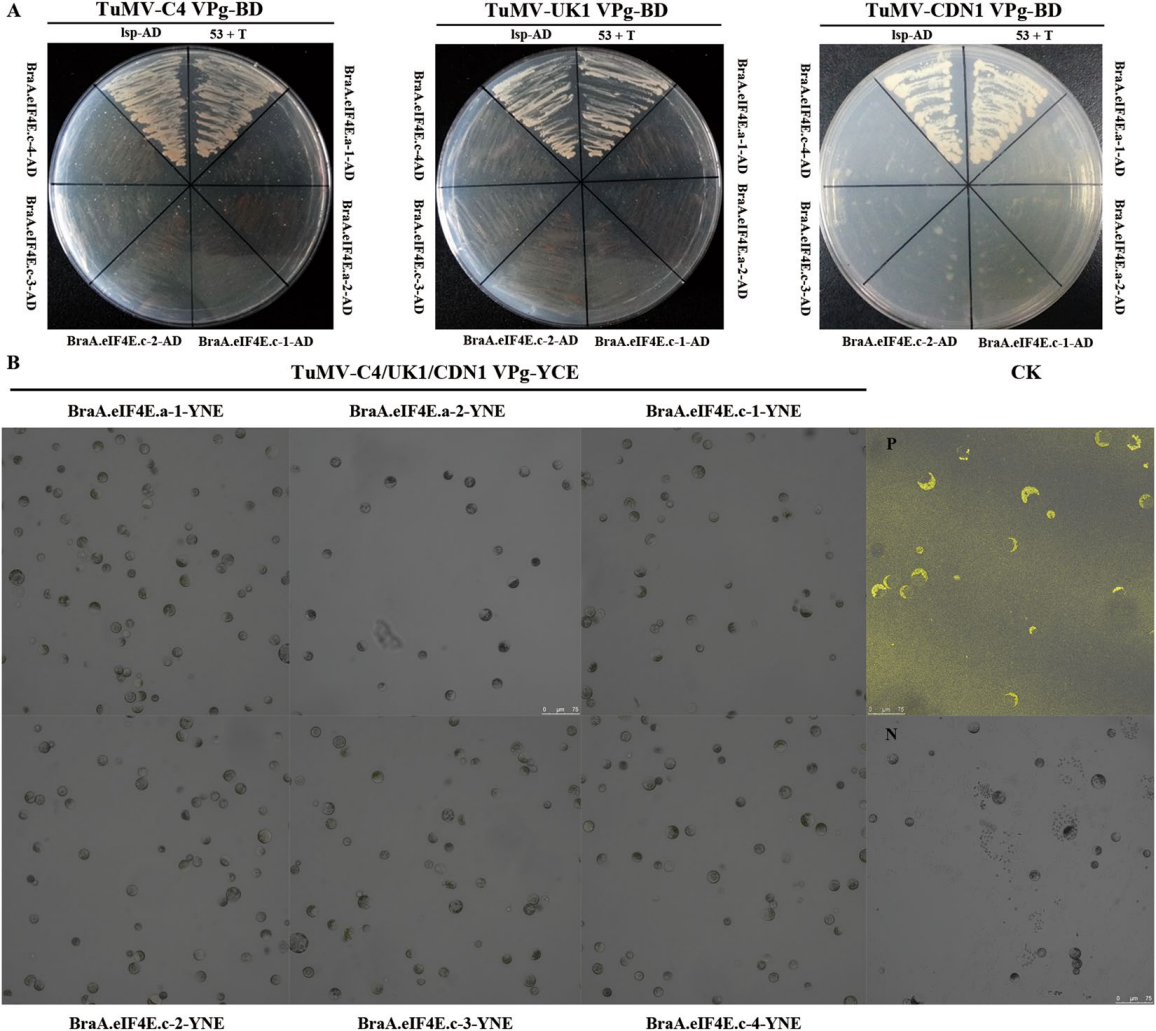
TuMV C4, TuMV UK1, and TuMV CDN1 VPgs do not interact with *BraA*.*eIF4E*.*a* or *BraA*.*eIF4E*.*c*. (**A**) The interaction was confirmed by yeast two-hybrid (Y2H) assays. Negative control: the empty vectors pGADT7 and pGBKT7 (data not shown); positive controls: the murine p53 and SV40 large T antigen from the Matchmaker GAL4 two-hybrid system 3; TuMV-VPg and *Arabidopsis* eIF(iso)4E (*lsp*); assay controls: each partner and empty vector (data not shown). (**B**) The interactions were confirmed by bimolecular fluorescence complementation (BiFC). P: positive controls (the combination of bZIP63YN and bZIP63YC); N: negative controls (YNE-empty and YCE-empty vectors); the assay controls: each partner and empty vectors (data not shown).

The results from the Y2H assays suggested that the positive control murine p53 interacts with the SV40 large T antigen, and the TuMV-C4/UK1/CDN1 VPgs could interact with the positive control LSP [*Arabidopsis* eIF(iso)4E], but not with BraA.eIF4E.a-1, BraA.eIF4E.a-2, BraA.eIF4E.c-1, BraA.eIF4E.c-2, BraA.eIF4E.c-3, or BraA.eIF4E.c-4 (Fig. 1A). BiFC was also used in *B*. *rapa* protoplast cells to study protein-protein interactions, and the results corroborated those generated by the Y2H assay (Fig. 1B). The empty vector pGADT7 did not interact with the empty vector pGBKT7, and each partner also did not interact with the empty vectors (data not shown).

### Different TuMV isolates interact with different eIF(iso)4Es in *B*. *rapa*

Compared with the eIF4E genes, the eIF(iso)4E genes were well conserved and showed a low degree of variability. Five different *BraA*.*eIF(iso)4E*.*a* alleles were identified, namely, *BraA*.*eIF(iso)4E*.*a-1* (80122-CDS, retaining the entire intron 1, resulting in a premature stop codon at position 234 bp), *BraA*.*eIF(iso)4E*.*a-2* (80122-CDS, with an extra G at the end of exon 1 that is predicted to result in a premature stop codon), *BraA*.*eIF(iso)4E*.*a-3* (80425), *BraA*.*eIF(iso)4E*.*a-4* (80186), and *BraA*.*eIF(iso)4E*.*a-5* (R-o-18). The sequences from 80425, 80186, and R-o-18 were also highly similar, and thus 80425 and 80186 were selected as representative lines for further investigation. Two different *BraA*.*eIF(iso)4E*.*c* alleles were also detected, namely, *BraA*.*eIF(iso)4E*.*c-1* (80122, 80425, 80186, 80124, BP058, 2079, and Chiifu) and *BraA*.*eIF(iso)4E*.*c-2* (R-o-18).

**Figure 2:**
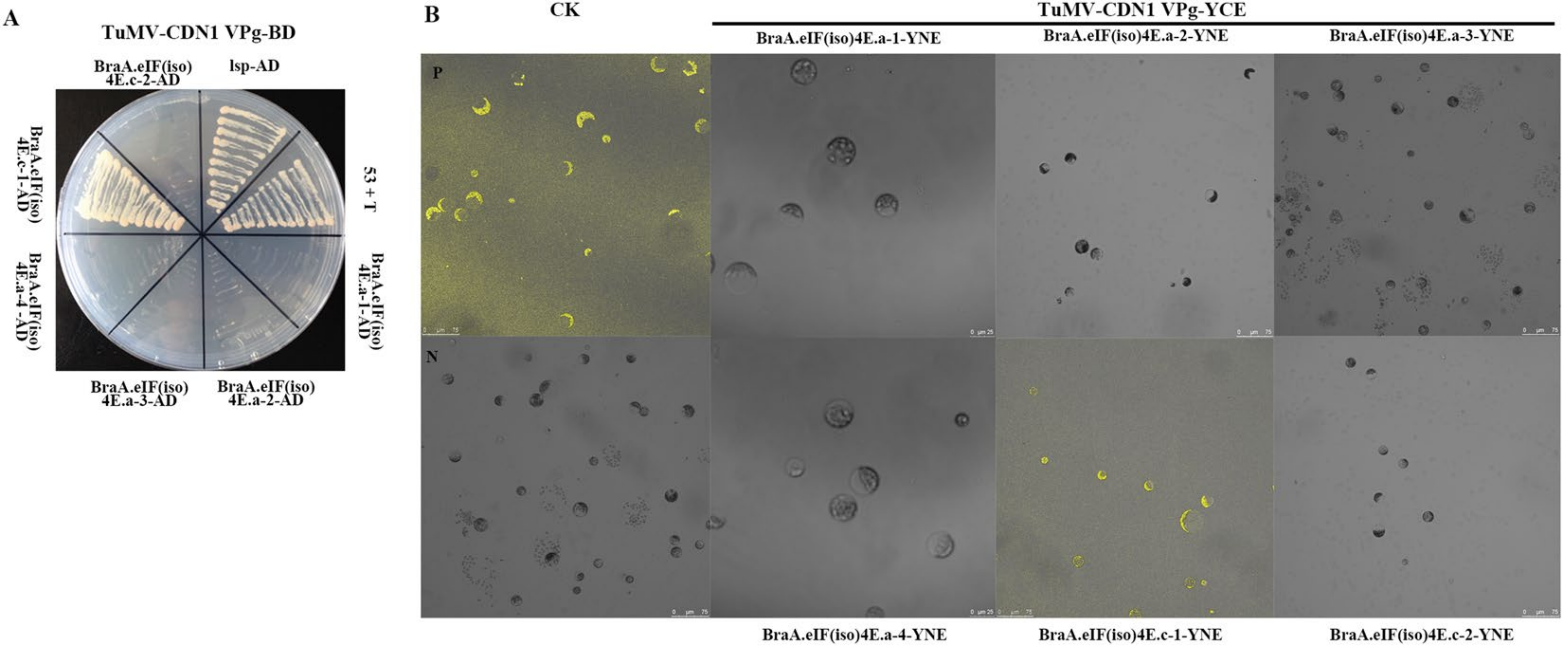
TuMV CDN1 VPgs interacts with *BraA*.*eIF(iso)4E*.*c*, but not with *BraA*.*eIF(iso)4E*.*a*. (**A**) The results are from the Y2H. Negative control: the empty vectors pGADT7 and pGBKT7 (data not shown); positive controls: the murine p53 and SV40 large T antigen from the Matchmaker GAL4 two-hybrid system 3; TuMV-VPg and *Arabidopsis* eIF(iso)4E (*lsp*); assay controls: each partner and empty vector (data not shown). (**B**) Verification of the results using BiFC. P: positive controls (the combination of bZIP63YN and bZIP63YC); N: negative controls (YNE-empty and YCE-empty vectors); each partner and empty vector were used as controls (data not shown).

The Y2H and BiFC analyses suggested that TuMV-CDN1 VPg largely interacts with BraA.eIF(iso)4E.c-1, but not with BraA.eIF(iso)4E.c-2 or BraA.eIF(iso)4E.a-1, BraA.eIF(iso)4E.a-2, BraA.eIF(iso)4E.a-3, or BraA.eIF(iso)4E.a-4 (Fig. 2A,B). We previously showed that TuMV-C4 VPg interacts with BraA.eIF(iso)4E.a, but not with BraA.eIF(iso)4E.c, whereas TuMV-UK1 VPg only interacts with BraA.eIF(iso)4E.c, but not BraA.eIF(iso)4E.a^53^. In *Arabidopsis*, TuMV VPg could only interact with LSP [*A*. *thaliana* harbors a single copy of *eIF(iso)4E*]. However, in *B*. *rapa* three *eIF(iso)4E* copies and different TuMV isolates were able to interact with various eIF(iso)4Es.

### Specific single nucleotide polymorphisms (SNPs) affect the interaction between eIF(iso)4E and VPg

**Figure 3:**
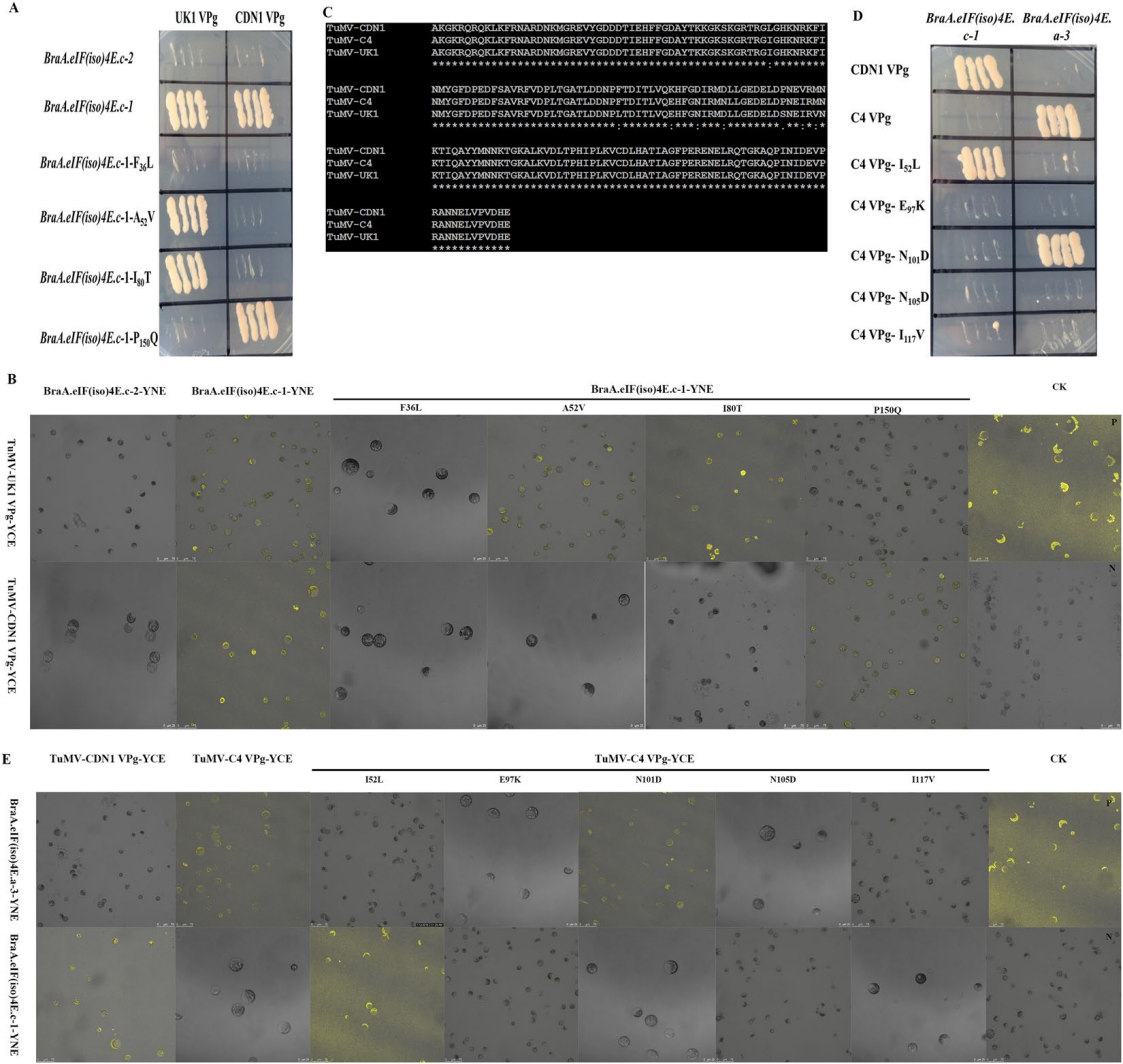
Specific SNPs affect the interaction between eIF(iso)4E and TuMV VPg. Variations between BraA.eIF(iso)4E.c-1 and BraA.eIF(iso)4E.c-2 could affect the interaction as confirmed by Y2H (**A**) and by BiFC. (**B**,**C**) Multiple sequence alignment of TuMV C4 VPg TuMV UK1 VPg and TuMV CDN1 VPg. Five amino acid substitutions were identified between TuMV C4 and CDN1, whereas four amino acid substitutions were detected between TuMV C4 and UK1. Variations between TuMV-C4 VPg and TuMV-CDN1 VPg could affect the interaction as indicated by Y2H (**D**) and by BiFC. (**E**) Y2H: negative control, the empty vectors pGADT7 and pGBKT7 (data not shown); positive controls, the murine p53 and SV40 large T antigen from the Matchmaker GAL4 two-hybrid system 3; TuMV-VPg and *Arabidopsis* eIF(iso)4E (*lsp*); assay controls: each partner and empty vector (data not shown). BiFC: P-positive controls (the combination of bZIP63YN and bZIP63YC); N-negative controls (YNE-empty and YCE-empty vectors); the assay controls were each partner and empty vectors (data not shown).

Some amino acid changes seem to be involved in strain-specific interactions between eIF(iso)4E and TuMV VPg. The Y2H and BiFC analysis indicated that TuMV-UK1/CDN1 VPg could interact with BraA.eIF(iso)4E.c-1 (Chiifu) but not with BraA.eIF(iso)4E.c-2 (R-o-18). Between *BraA*.*eIF(iso)4E*.*c-1* (Chiifu) and *BraA*.*eIF(iso)4E*.*c-2* (R-o-18), five differing bases (nt T_106_C, C_155_T, T_239_C, C_449_A, and C_546_T) were identified (Table S4), which were predicted to result in the substitution of four amino acids (F_36_L, A_52_V, I_80_T, and P_150_Q) (Table 4). Thus, primers were designed at the four loci, and site-directed mutagenesis (using *eIF(iso)4E* of Chiifu as template) was successfully implemented based on the overlap-extension PCR to detect which locus was essential for the interactions. Y2H and BiFC analyses indicated that the amino acid substitution F_36_L (nt T_106_C) in BraA.eIF(iso)4E.c played a critical role in the interaction between TuMV-UK1 VPg and BraA.eIF(iso)4E.c-1 (Chiifu), and the amino acids A_52_V (nt C_155_T), I_80_T (nt T_239_C), and P_150_Q had little influence (Fig. 3A,B). Compared to TuMV-UK1 VPg, TuMV-CDN1 VPg in the Y2H and BiFC assays performed differently in the interaction: amino acids F_36_L (nt T_106_C), A_52_V (nt C_155_T), and I_80_T (nt T_239_C) in *BraA*.*eIF(iso)4E*.*c* were the key elements; and P_150_Q did not play an essential role in the observed interaction (Fig. 3A,B). Taken together, the amino acid F_36_L (nt T_106_C) in BraA.eIF(iso)4E.c is a key site of the protein that generally affects the interaction between BraA.eIF(iso)4E.c-1 and TuMV-UK1/CDN1 VPg.

TuMV-C4 VPg could interact with BraA.eIF(iso)4E.a-3 (80425); while TuMV-CDN1 VPg could not. Sequence analysis of TuMV-C4 VPg and TuMV-CDN1 VPg identified five base/amino acid changes (nts A_154_C, G_289_A, A_301_G, A_313_G, and A_349_G; aas I_52_L, E_97_K, N_101_D, N_105_D, and I_117_V) (Fig. 3C). Five mutants (using TuMV-C4 *VPg* as a template) were obtained by overlap-extension PCR. The Y2H and BiFC analyses indicated that the interaction between TuMV-C4 VPg and BraA.eIF(iso)4E.a-3 (80425) was mediated by the amino acid substitutions I_52_L, E_97_K, and N_105_D of TuMV VPg, and the amino acid substitution N_101_D and I_117_V did not affect this particular interaction (Fig. 3D,E). Compared to BraA.eIF(iso)4E.a-3 (80425), BraA.eIF(iso)4E.c-1 (Chiifu) exhibited differences in this interaction. In the trials, the amino acid substitution I_52_L was essential for the interaction, whereas the other four sites did not affect the interaction (Fig. 3D,E). Taken together, the amino acid substitution I_52_L in TuMV-C4 VPg plays a critical role in the interaction between BraA.eIF(iso)4E.a and TuMV VPg. Therefore, various amino acids in eIF(iso)4E were essential to the interaction, whereas a few amino acids in TuMV VPg were also significant in the interaction.

### Analysis of the mechanism underlying the interaction of TuMV VPg-eIF(iso)4E

**Figure 4:**
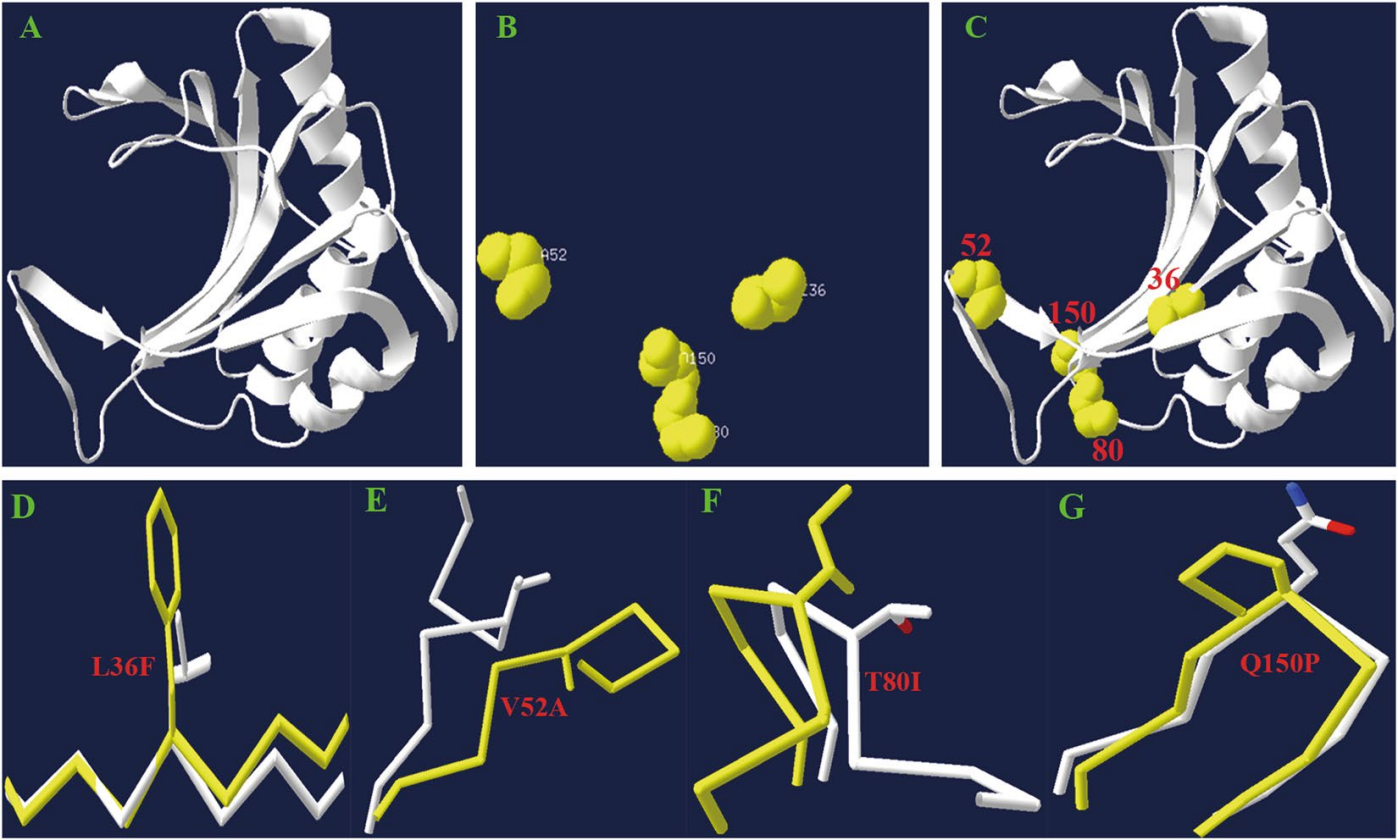
Analysis of BraA.eIF(iso)4E.c-1 protein domain tertiary structure. (**A**) The three-dimensional structural model was built based on the wheat eIF(iso)4E protein. (**B**) Four amino acid variations were detected between the BraA.eIF(iso)4E.c-1 and BraA.eIF(iso)4E.c-2 proteins. (**C**) The location of the three different amino acid variations is depicted in the structural model of the entire protein. (**D**) The 36^th^ amino acid changed from phenylalanine to leucine. (**E**) The 52^th^ amino acids changed from valine to alanine. (**F**) The 80^th^ changed from isoleucine to threonine. (**G**) The 150^th^ changed from proline to glutamine.

Amino acid sequence alignment of BraA.eIF(iso)4E.c-1 (Chiifu) and BraA.eIF(iso)4E.c-2 (R-o-18) identified four substitutions: Phe/Leu-36, Ala/Val-52, Ile/Thr-80, and Pro/Gln-150 (Table 4). Furthermore, a three-dimensional (3D) structural model of the BraA.eIF(iso)4E.c-1 protein was constructed (Fig. 4A) to identify the special conformation of the four sites. The 36^th^ amino acid in BraA.eIF(iso)4E.c is highly conserved and is pivotal in establishing the structural stability of the protein. The amino acid Phe is highly conserved in plant eIF4Es and eIF(iso)4Es, whereas in human and yeast, this site is occupied by a Leu residue^54^. The 36^th^ amino acid in BraA.eIF(iso)4E.c changed from Phe to Leu, and Phe is an aromatic amino acid that contains a benzene ring. However, Leu is an aliphatic amino acid that is linear and does not contain a benzene ring (Fig. 4B). Thus, the two amino acid substitutions may influence the biochemical functions and the structure of the protein. Compared to the 36^th^ amino acid, the 52^th^, 80^th^, and 150^th^ amino acids are not conserved in the protein. The 52^th^ amino acids changed from Val to Ala, the 80^th^ changed from Ile to Thr, and the 150^th^ changed from Pro to Gln, which are all aliphatic amino acids (Fig. 4B).

The structure of the BraA.eIF(iso)4E protein includes eight *β*-strands, three *α*-helices, and three extended loops. There is a large cavity at its cap-binding site that undergoes conformational changes in the cap-binding loops. The 36^th^ amino acid is located in the middle of the first *β*-strands region and may play an essential role in protein structure and function (Fig. 4A). In addition, the 36^th^ amino acid is located in the cap-free structure of BraA.eIF(iso)4E protein, thereby suggesting that it plays an essential role in the allosteric regulation of the BraA.eIF(iso)4E protein.

## Discussion

In a previous study, the *BraA*.*eIF4Es* and *BraA*.*eIF(iso)4Es* from the *B*. *rapa* ‘RLR22’ line could not interact with the TuMV isolates^51^. The two copies of *eIF4E* (*BraA*.*eIF4E*.*a* and *BraA*.*eIF4E*.*c*) and two copies of *eIF(iso)4E* [*BraA*.*eIF(iso)4E*.*a* and *BraA*.*eIF(iso)4E*.*c*] were transformed into Col-0::*dSpm*, which had a transposon knocked out of the *eIF(iso)4E* gene. However, this resulted in a change from complete susceptibility to complete resistance to TuMV, and all four *Brassica* transgenes complemented the *A*. *thaliana eIF(iso)4E* knockout. These changes conferred susceptibility to both mechanical and aphid challenge with TuMV^51,52^. In this study, the Y2H and BiFC assays also showed that the TuMV-C4/UK1/CDN1 isolates did not interact with *BraA*.*eIF4Es*, but rather with *BraA*.*eIF(iso)4Es*. The interaction of the eIFs and TuMV VPgs differed between *B*. *rapa* and *A*. *thaliana*. *BraA*.*eIF4Es* and *BraA*.*eIF(iso)4Es* from the *B*. *rapa* ‘RLR22’ line could not interact with the TuMV isolates *in vitro*, but could interact with the TuMV isolates in the Col-0::*dSpm*, which was somewhat misleading. Genomic analyses of diploid *B*. *rapa* have indicated that it evolved from a hexaploid ancestor and then underwent a whole genome triplication event^55^, which resulted in more gene copies in *B*. *rapa* than in *A*. *thaliana*. These genomic changes have formed more complex compounds that are related to the TuMV infection process, and the same genes induce different results in *B*. *rapa* and *A*. *thaliana*.

Thus, different parts of eIF(iso)4E and various amino acids in VPg influence this particular interaction. Sequence comparison between BraA.eIF(iso)4E.a and BraA.eIF(iso)4E.c identified various amino acid substitutions that may affect the interactions (Fig. S1). Comparison of the TuMV-C4 and TuMV-UK1 VPgs identified four amino acids substitutions, namely, F_89_L, N_105_D, P_114_S, and M_119_V (Fig. 3C). These amino acid changes, particularly those involving residues 89 and 114, may influence the interaction. Thus, it is possible that different strains of TuMV interact with various eIF(iso)4E proteins to influence protein translation. Some amino acids indicated evidence of positive selection, which may have contributed to virus resistance in eIF4E and eIF(iso)4E. For example, in wheat, the G_107_R substitution in the cap-binding pocket plays a key role in both VPg interactions and cap-binding, whereas the L_79_R change that is located within an external loop influences VPg, but not cap-binding^56^.

BraA.eIF(iso)4E.a interacted with the TuMV-C4 VPg, whereas BraA.eIF(iso)4E.a-1 and −2 in 80122 showed loss of function and could not interact with the TuMV-C4 VPg. Thus, *BraA*.*eIF(iso)4E*.*a-1* and *−2* are resistant alleles for TuMV-C4, and the deleted parts of their proteins (from 70th to 200th amino acids) are essential to this particular interaction. TuMV-UK1 VPg interacted with BraA.eIF(iso)4E.c-1, but not with BraA.eIF(iso)4E.c-2. Four amino acid variations (L_36_F, V_52_A, T_80_I, and Q_150_P) between BraA.eIF(iso)4E.c-1 and BraA.eIF(iso)4E.c-2 influence this interaction. Thus, *BraA*.*eIF(iso)4E*.*c* contains a resistance locus for TuMV-UK1. In *A*. *thaliana*, structural data implicate Trp-46 and Trp-92 in eIF(iso)4E in cap recognition and when Trp-46 or Trp-92 is changed to Leu, eIF(iso)4E loses the ability to form a complex with both VPgs^57^. eIF4E and eIF(iso)4E belong to class I of the eIF4E family, and the novel cap-binding protein nCBP belongs to class II^58^. Members from class I have conserved Trp-43 and Trp-56, while those from class II have both residues substituted by Trp or Phe^56^. The co-evolution of the VPg of TuMV and *eIF(iso)4E* in *B*. *rapa* may have resulted in variations in both proteins. Further investigation into the co-evolutionary relationship between TuMV and *B*. *rapa* suggests that the amino acids of eIF(iso)4E that influence its interaction with the VPgs of different TuMV isolates are highly variable.

Accordingly, the TuMV isolate resistance loci could be screened to identify resistance genes and could be cloned to generate plants with broad-spectrum resistance. Furthermore, the 3D structural model of the BraA.eIF(iso)4E.c protein indicates that the 36^th^ amino acid is highly conserved and plays an important role in stabilizing the protein structure. However, the main purpose of building a structural protein model was to provide the location of the mutation in a 3D structural context. This may provide information on whether the mutation is buried within the hydrophobic core, lying on the surface, located near an active site, proximal to an interface, or close to a site of post-translational modification. This model may also be used as a guide in predicting the effect of specific mutations on protein function.

In *Arabidopsis*, TuMV VPg can only interact with LSP [eIF(iso)4E]. Mutations involving *LSP* (i.e., *lsp* mutants) exhibit premature termination, thereby conferring loss of function and resistance to TuMV^18,48^. In *B*. *rapa*, *retr02* [*BraA*.*eIF(iso)4E*.*a*] has been identified as a recessive resistance gene for TuMV C4^16,59^. The product of the resistance gene *retr02* is also involved in premature protein termination and is thus unable to interact with TuMV C4 VPg, thereby resulting in resistance to TuMV C4^52^. Thus, the introduction of mutations in the *BraA*.*eIF(iso)4E*.*a* gene may confer plant resistance to TuMV C4, e.g., *Retr02*, and mutations in the *BraA*.*eIF(iso)4E*.*c* gene induce resistance to TuMV UK1. In addition, mutations in both *BraA*.*eIF(iso)4E*.*a* and *BraA*.*eIF(iso)4E*.*c* confer resistance to both TuMV C4 and TuMV UK1. It is also possible to determine whether TuMV isolates affect such mutants; and when these are resistant, the range of activity (broad resistance). This strategy could be used to create new varieties that may be used in breeding as well as in marker-assisted selection.

## Materials and Methods

### Plant materials and TuMV isolates

The following *B*. *rapa* accessions were used in this study: 80122 (BP8407), 80425 (Ji Zao Chun), 80186 (Er Qing), 80124(89B), Chiifu, 2079, R-o-18, and BP058, which are highly inbred lines. The lines 80122, 80186, 80124, Chiifu, 2079, and BP058 are resistant to TuMV C4, whereas 80425 and R-o-18 are susceptible to the TuMV isolate^16,59^. The CDS sequences of eIF4E and eIF(iso)4E genes from 8 lines were submitted to GenBank (Table 5).

**Table 5:** The accession numbers of eIF4E and eIF(iso)4E genes from 8 lines.

Gene ID	80124	BP058	2079	80186	80122	80425	Chiifu	R-o-18
eIF4E.a	MH614206	MH614207	MH614208	MH614209	MH614210	MH614211	MH614212	MH614213
eIF4E.c	MH614218	MH614220	MH614217	MH614219	MH614214	MH614216	MH614215	MH614221
eIF(iso)4E.a	MH614224	MH614225	MH614226	MH614228	MH614222	MH614227	MH614229	MH614230
					MH614223			
eIF(iso)4E.a	MH614236	MH614235	MH614232	MH614237	MH614234	MH614233	MH614231	MH614238

Three representative pathotypes, namely TuMV isolates C4 [HQ46217] from China, CDN1 [D83184] from Canada, and UK1 [NC_002509] from the UK, were maintained in the susceptible mustard (*B*. *juncea*) cultivar Tender Green.

### Identification of eIF4E and its isoform eIF(iso)4E in *B*. *rapa*

The *B*. *rapa* genome has been sequenced, and the *Brassica* database (BRAD) includes predicted genes and associated annotations (InterPro, KEGG2, SWISS-PROT), *B*. *rapa* genes orthologous to those in *A*. *thaliana*, and genetic markers and maps for *B*. *rapa*.

Sequences representing the complete set of eIF4E and eIF(iso)4E genes in *A*. *thaliana* were acquired from The Arabidopsis Information Resource (TAIR) database (www.arabidopsis.org) and were used to search the data from the *B*. *rapa* ssp. *pekinensis* cv. Chiifu genome V1.5 and its set of annotated genes (http://Brassicadb.org) for homologous genes. Estimation of the number of eIF4E and eIF(iso)4E genes in the genome of *B*. *rapa* was conducted by analysis of expressed sequence tag (EST) data downloaded from the NCBI EST database and mRNA sequencing data (unpublished data). Full-length eIF4E and eIF(iso)4E protein sequences of *A*. *thaliana* genes were retrieved from the TAIR database and the UniProt protein database (www.uniprot.org).

### Cloning and sequencing of eIF4E and its isoform eIF(iso)4E

Total RNAs were extracted from the leaves of the *B*. *rapa* lines (80122, 80425, 80186, 80124, Chiifu, 2079, R-o-18, and BP058) using TRIzol reagent (Invitrogen, Carlsbad, CA, USA), and first-strand cDNA was synthesized with a polydT primer using a Prime Script^TM^ RT-PCR kit (TaKaRa, Dalian, China) according to the manufacturer’s instructions. The cDNAs were used as templates for PCR.

Generic primers (Table S5) were designed using the eIF4E gene reference sequence from the *B*. *rapa* genome, which encompassed the majority of the ORF. PCR was performed on the cDNAs using KOD Hot Start DNA polymerase (TOYOBO, Osaka, Japan). The PCR products were sequenced to analyze the allelic variability of the genes.

### Y2H

Interactions between proteins were assayed with a Gal4-based Y2H system, as described by the manufacturer (Clontech, Mountain View, CA, USA). Yeast strains and plasmid vectors were obtained from Clontech Laboratories (Clontech). A bait plasmid, pGBKT7, was used to fuse the VPg to the DNA-binding domain of BD. A prey plasmid, pGADT7, was used to express the eIF4E genes (Clontech). Gene-specific primers were designed to introduce restriction enzyme sites (Table S6). The DNA sequences encoding VPg from TuMV-C4 and TuMV-UK1 were amplified using forward primer Bio120213 (*Nde*I site) and reverse primer Bio120214 (*Xma*I site). *BraA*.*eIF4E*.*a* was amplified using forward primer Bio120850 (*Eco*RI site) and reverse primer Bio120851 (*Xho*I site), *BraA*.*eIF4E*.*c* was amplified using forward primer Bio120852 (*Eco*RI site) and reverse primer Bio120853 (*Xho*I site), *BraA*.*eIF(iso)4E*.*c* was amplified using forward primer Bio120854 (*Eco*RI site) and reverse primer Bio120855 (*Xho*I site), and *BraA*.*eIF(iso)4E*.*a* was amplified using forward primer Bio120075 (*Eco*RI site) and reverse primer Bio120076 (*Xho*I site). The eIF(iso)4E sequences from *Arabidopsis* Col-0 were amplified using forward primer Bio120582 (*Eco*RI site) and reverse primer Bio120583 (*Xho*I site), and cloned into pGADT7 as a positive control for the interaction in the yeast two-hybrid assay. The amplified fragments were digested with *Eco*RI/*Xho*I and *Nde*I/*Xma*I and cloned into the corresponding restriction sites of pGADT7 and pGBKT7, respectively. All constructs were confirmed by sequencing.

The Matchmaker GAL4 two-hybrid system (Clontech) was used according to the manufacturer’s protocols. pGADT7:4E and pGBKT7:VPg constructs were transformed into AH109 yeast strains. After yeast transformation, colonies were grown on various selective media lacking leucine, tryptophan, histidine, and adenine (SD-LW/SD-LWH/SD-LWHA). Plates were incubated at 30 °C and growth was checked 3–5 days after inoculation. Each assay was performed in triplicate. The empty vectors pGADT7 and pGBKT7 were used as negative controls; the interaction between murine p53 and SV40 large T antigen (controls from the Matchmaker GAL4 two-hybrid system 3) was used as a positive control; and TuMV-VPg and *Arabidopsis* eIF(iso)4E (*LSP*) were also used as positive controls. In addition, each partner and empty vectors were used as assay controls.

### BiFC

Molecular techniques were performed using standard protocols^60–62^. The *eIF(iso)4E* genes were amplified using the primers above, which contained the *BamH*I and *Xho*I sites, and the *eIF(iso)4E* genes PCR products and pSPYNE empty vector were digested by *BamH*I and *Xho*I. Then, the recombinant vector eIF(iso)4E*-*pSPYNE was constructed using T_4_ ligase. Similarly, the TuMV VPg genes were amplified using specific primers (Table S7), which contained the *Cla*I and *Xho*I sites, and the TuMV VPg gene PCR products and pSPYCE empty vector were digested by the *Cla*I and *Xho*I sites. The primer pair Bio120901/Bio120902 was used to amplify *BraA*.*eIF4E*.*a*, Bio120903/Bio120904 for *BraA*.*eIF4E*.*c*, Bio120905/Bio120906 for *BraA*.*eIF(iso)4E*.*c*, Bio120907/Bio120908 for *BraA*.*eIF(iso)4E*.*a*, Bio120909/Bio120910 for *LSP*, and Bio120911/Bio120912 for *VPg*. The recombinant vector TuMV VPg-pSPYCE was constructed using T_4_ DNA ligase. The recombinant vectors were confirmed by sequencing. Each experiment was performed in triplicate. The positive controls included the combination of bZIP63YN and bZIP63YC, and the negative controls were the YNE-empty and YCE-empty vectors. In addition, each partner and empty vector was used as controls.

The *B*. *rapa* protoplasts were prepared and cultured as described elsewhere^62^. The fresh leaves were obtained from Chinese cabbage plants at the four-leaf stage. The reagents used in the assays were 1.5% cellulase R10; 0.4% mecerozyme R10; 0.4 M D-mannitol; 20 mM KCl; 20 mM MES (pH 5.7); 10 mM CaCl_2_; 0.1% BSA; 5 mM β-mercaptoethanol, and the assays were conducted as indicated in the protocol^62^. The recombinant vectors [eIF(iso)4E*-*pSPYNE, TuMV VPg-pSPYCE, bZIP63YN, bZIP63YC, YNE-empty and YCE-empty, constructed in BiFC assays] were transfected into the protoplasts, the plant cells were cultured in the dark, and the fluorescence signals were assessed using a laser confocal scanning microscope after 24-h.

### Equipment and settings

The three-dimensional (3D) structural of BraA.eIF(iso)4E.c-1 protein was modeled by Phyre2^63^, and the model was displayed by Swiss-PdbViewer^64^.

In BiFC assays, the fluorescence signals were assessed in the protoplasts by laser confocal scanning microscope (LCSM)^65^. Fluorophores Compatible with the ZOE Fluorescent Cell Imaging System, YFP channel, excitation: 480/17 nm; emission: 517/23 nm; CytoTrack YFP 511/525; VivaFix 498/521 Cell Viability Assay. And the images were combined by Adobe Photoshop CS6 (https://helpx.adobe.com/creative-suite/kb/cs6-install- instructions.html).

### Accession numbers

HQ446217 (TuMV C4), D83184 (TuMV CDN1), NC_002509 (TuMV UK1).

## Electronic supplementary material

Supplementary Information
