# Supplementary material for: Variability in eukaryotic initiation factor iso4E in Brassica rapa influences interactions with the viral protein linked to the genome of Turnip mosaic virus
Source: Sci Rep. 2018 Sep 11;8:13588. doi: 10.1038/s41598-018-31739-1 (PMC6134127; doi:10.1038/s41598-018-31739-1)
Supplement: Supplementary file 1 — Supplementary Information [file 41598_2018_31739_MOESM1_ESM.docx]

**Variability in eukaryotic initiation factors iso4E in *Brassica rapa* influences interactions with the viral protein linked to the genome of *Turnip mosaic virus***

Guoliang Li^†^, Wei Qian^†^, Shujiang Zhang, Shifan Zhang, Fei Li, Hui Zhang, Zhiyuan Fang, Jian Wu, Xiaowu Wang & Rifei Sun*

Institute of Vegetables and Flowers, Chinese Academy of Agricultural Sciences, Beijing, Zhongguancun, Nandajie No. 12, Haidian District, Beijing 100081, People’s Republic of China

*For correspondence (e-mails: sunrifei@caas.cn).

†These two authors contributed equally to this work.


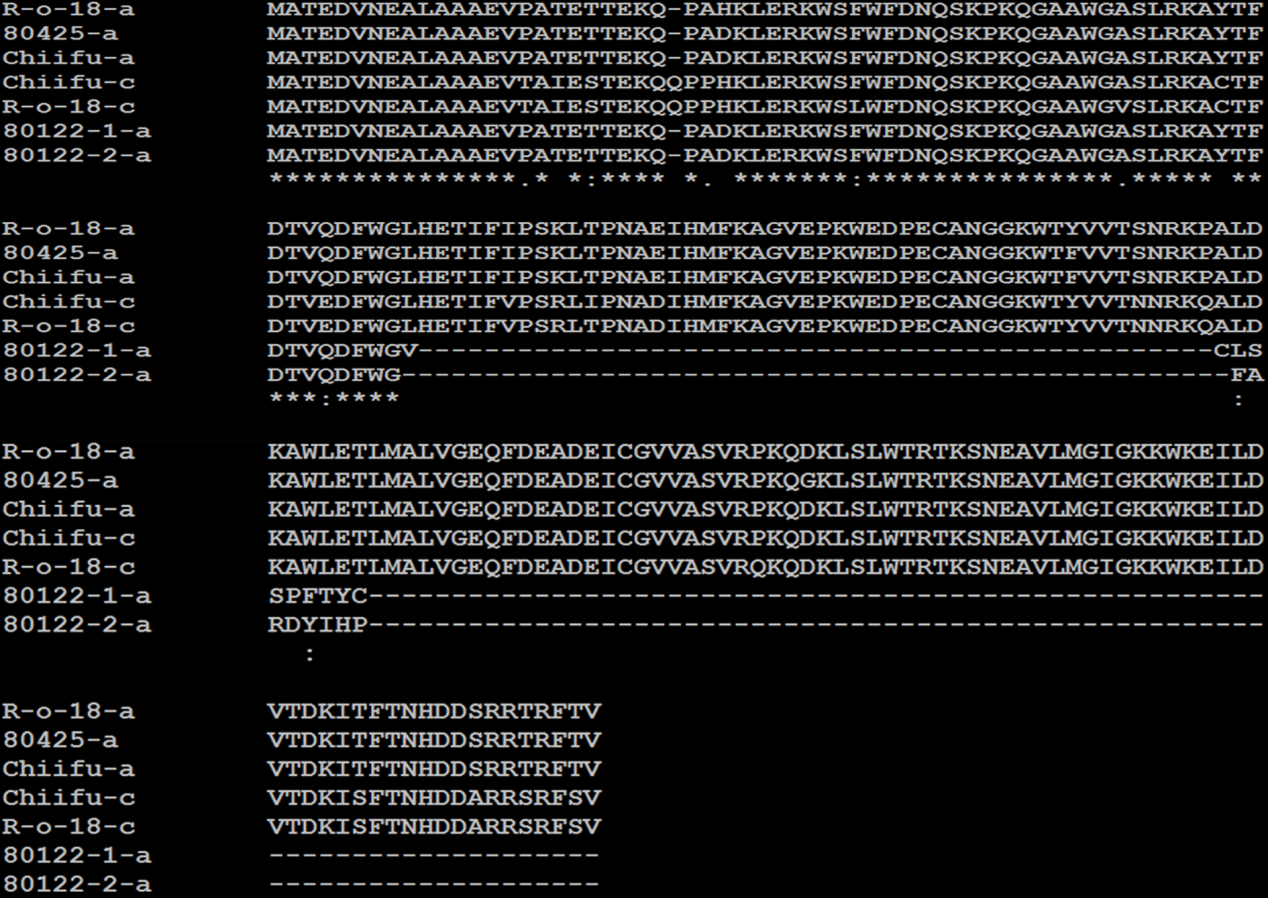


**Fig. S1.** Multiple sequence alignment of *BraA.eIF(iso)4E.a* and *BraA.eIF(iso)4E.c*. -a: *BraA.eIF(iso)4E.a*; -c: *BraA.eIF(iso)4E.c*. *BraA.eIF(iso)4E.a*has two splice variants in the 80122 line, namely, 80122-1 (which retains the entire intron 1 and is predicted to result in a premature stop codon at position 234 bp) and 80122-2 (with an extra G at the end of exon 1 and is predicted to generate a premature stop codon).

**Table S1.** Base changes in *BraA.eIF4E.a*

| Line | 34 | 62 | 98 | 119 | 164 | 300 | 335 | 444 | 525 | 564 | 638 |
| --- | --- | --- | --- | --- | --- | --- | --- | --- | --- | --- | --- |
| 80124 | C | T | A | T | T | T | A | C | A | T | A |
| BP058 | C | T | A | T | T | T | A | C | A | T | A |
| 2079 | C | T | A | T | T | T | A | C | A | T | A |
| 80186 | C | T | G | T | C | T | A | C | A | T | G |
| 80122 | G | C | A | C | T | C | G | T | G | G | A |
| 80425 | G | C | A | C | T | C | G | C | A | T | A |
| Chiifu | G | C | A | C | T | C | G | C | A | T | A |
| R-o-18 | G | C | A | C | T | C | G | C | A | T | A |

**Table S2.** Base changes in *BraA.eIF4E.c*

| Line | 68 | 103 | 104 | 105 | 133 | 134 | 162 | 198 | 273 | 360 | 393 | 399 | 405 | 429 | 462 | 495 | 545 | 602 |
| --- | --- | --- | --- | --- | --- | --- | --- | --- | --- | --- | --- | --- | --- | --- | --- | --- | --- | --- |
| 80122 | A | A | C | C | G | G | C | C | C | A | T | T | T | G | C | T | G | A |
| Chiifu | A | A | C | C | G | G | C | C | C | A | T | T | T | G | C | T | G | A |
| 80186 | A | G | C | T | G | G | C | T | T | A | T | A | A | A | C | T | A | G |
| 80124 | G | G | C | T | G | G | G | T | T | A | T | A | A | A | C | T | G | G |
| BP058 | A | G | C | T | G | G | C | T | T | A | T | A | A | A | C | T | A | G |
| R-o-18 | A | G | C | T | G | G | C | T | T | A | T | A | A | A | C | T | A | G |
| 80425 | A | G | T | T | A | C | C | C | C | G | C | T | A | G | T | C | G | A |
| 2079 | A | G | T | T | A | C | C | C | C | G | C | T | A | G | T | C | G | A |

**Table S3.** Base changes in *BraA.eIF(iso)4E.a*

| Line | 79 | 202 | 309 | 323 | 330 | 420 | 426 | 455 | 586 |
| --- | --- | --- | --- | --- | --- | --- | --- | --- | --- |
| 80122-1 | G | G | - | - | - | - | - | - | - |
| 80122-2 | G | G | - | - | - | - | - | - | - |
| 80124 | G | G | - | - | - | - | - | - | - |
| BP058 | G | G | - | - | - | - | - | - | - |
| 2079 | G | G | - | - | - | - | - | - | - |
| 80186 | G | - | C | T | T | C | G | A | C |
| Chiifu | G | - | C | T | T | C | G | A | C |
| 80425 | G | - | C | T | T | C | G | G | C |
| R-o-18 | C | - | G | A | C | T | C | A | A |

*Note: The BraA.eIF(iso)4E.a has two splice variants in 80122 line, which respectively are 80122-1 (retained the whole of intron 1, resulting in a premature stop codon at position 234 bp) and 80122-2 (with an extra G at the end of exon 1, resulting a premature stop codon)* (Nellist *et al*., 2014)*.*

**Table S4.** Base changes in *BraA.eIF(iso)4E.c*.

| Line | 106 | 155 | 239 | 449 | 546 |
| --- | --- | --- | --- | --- | --- |
| 80186 | T | C | T | C | C |
| 80124 | T | C | T | C | C |
| BP058 | T | C | T | C | C |
| 80122 | T | C | T | C | C |
| 80425 | T | C | T | C | C |
| 2079 | T | C | T | C | C |
| Chiifu | T | C | T | C | C |
| R-o-18 | C | T | C | A | T |

**Table S5.** Primers used for the amplification of the eIF4E and eIF(iso)4E genes

| Primers | eIF-Genes | 5’-3’ |
| --- | --- | --- |
| Bio120734 | *BraA.eIF4E.a* | ATGGCGGTAGAAGACACA |
| Bio120735 | *BraA.eIF4E.a* | TCAGGCAGTGTAAGCGCT |
| Bio120736 | *BraA.eIF4E.c* | ATGGCGGTAGAAGACACT |
| Bio120737 | *BraA.eIF4E.c* | CGGTGTAAGCGCTCTTCG |
| Bio120742 | *BraA.eIF(iso)4E.c* | ATGGCGACAGAGGATGT |
| Bio120743 | *BraA.eIF(iso)4E.c* | TCAGACACTAAATCGAC |
| Bio11535 | *BraA.eIF(iso)4E.a* | ATGGCGACAGAGGATGTG |
| Bio11536 | *BraA.eIF(iso)4E.a* | TCAGACAGTGAACCGAG |

**Table S6**. Primers used in Y2H

| Primers | eIF-Genes | 5’-3’ |
| --- | --- | --- |
| Bio120850 | *BraA.eIF4E.a* | GGAATTCATGGCGGTAGAAGACAC |
| Bio120851 | *BraA.eIF4E.a* | CCTCGAGGTCAGGCAGTGTAAGC |
| Bio120852 | *BraA.eIF4E.c* | GGAATTCATGGCGGTAGAAGACAC |
| Bio120853 | *BraA.eIF4E.c* | CCTCGAGGTCAAGCGGTGTAAGCGCTC |
| Bio120854 | *BraA.eIF(iso)4E.c* | GGAATTCATGGCGACAGAGGATGTGA |
| Bio120855 | *BraA.eIF(iso)4E.c* | CCTCGAGGTCAGACACTAAATCGAC |
| Bio120075 | *BraA.eIF(iso)4E.a* | GGAATTCCATGGCGACAGAGGATGTG |
| Bio120076 | *BraA.eIF(iso)4E.a* | CCGCTCGAGTCAGACAGTGAACCGAG |
| Bio120582 | *LSP* | GGAATTCATGGCGACCGATGATGTG |
| Bio120583 | *LSP* | CCTCGAGGTCAGACAGTGAACCGGC |
| Bio120213 | *VPg* | CCCATATGATGGCGAAAGGTAAGAGGC |
| Bio120214 | *VPg* | CCCCGGGTCACTCGTGGTCCACTGGGA |

**Note:** the underlines were the enzyme sites.

**Table S7**. Primers used in BiFC

| Primers | eIF-Genes | 5’-3’ |
| --- | --- | --- |
| Bio120901 | *BraA.eIF4E.a* | GGGATCCATGGCGGTAGAAGACAC |
| Bio120902 | *BraA.eIF4E.a* | CCTCGAGGTCAGGCAGTGTAAGC |
| Bio120903 | *BraA.eIF4E.c* | GGGATCCATGGCGGTAGAAGACAC |
| Bio120904 | *BraA.eIF4E.c* | CCTCGAGGTCAAGCGGTGTAAGCGCTC |
| Bio120905 | *BraA.eIF(iso)4E.c* | GGGATCCATGGCGACAGAGGATGTGA |
| Bio120906 | *BraA.eIF(iso)4E.c* | CCTCGAGGTCAGACACTAAATCGAC |
| Bio120907 | *BraA.eIF(iso)4E.a* | GGGATCCCATGGCGACAGAGGATGTG |
| Bio120908 | *BraA.eIF(iso)4E.a* | CCGCTCGAGTCAGACAGTGAACCGAG |
| Bio120909 | *LSP* | GGGATCCATGGCGACCGATGATGTG |
| Bio120910 | *LSP* | CCTCGAGGTCAGACAGTGAACCGGC |
| Bio120911 | *VPg* | CCATCGATATGGCGAAAGGTAAGAGGC |
| Bio120912 | *VPg* | CCTCGAGTCACTCGTGGTCCACTGGGA |

**Note:** the underlines were the enzyme sites.
